# A Real‐World Disproportionality Analysis of Avacopan in Anti‐Neutrophil Cytoplasmic Antibodies Associated Vasculitis: Insights From FDA Adverse Event Reporting System

**DOI:** 10.1002/prp2.70194

**Published:** 2025-11-17

**Authors:** Guojun Liang, Xiaofang Liu, Mengge Gao, Bo Yang, Yang Song, Qiong Liu

**Affiliations:** ^1^ Huadu District People's Hospital of Guangzhou Guangzhou Guangdong China

**Keywords:** avacopan, disproportionality analysis, FAERS, pharmacovigilance, post‐marketing safety

## Abstract

Avacopan, an oral C5a receptor antagonist approved for treating anti‐neutrophil cytoplasmic antibodies (ANCA) associated vasculitis, has established efficacy and short‐term safety from clinical trials, but its post‐marketing adverse events (AEs) in real‐world settings require further characterization. We conducted a retrospective analysis of the U.S. FDA Adverse Event Reporting System (FAERS) database from Q1 2022 to Q1 2025. After data cleaning, Avacopan‐related AEs were extracted, coded using MedDRA terminology, and analyzed via four signal detection methods; subgroup analyses by age, sex, and reporter type were performed. Among 3529 reports, significant disproportionality signals emerged for known AEs (e.g., hepatobiliary disorders, serious infections) and unexpected signals including venous thromboembolism, cholestatic jaundice, and alopecia. Most AEs occurred within the first 30 days of treatment, with variations observed by age and sex. This study provides the first FAERS‐based pharmacovigilance assessment of Avacopan, confirming known risks and identifying novel post‐approval safety signals, underscoring the need for close early‐treatment monitoring and personalized strategies. Further research is warranted to confirm emerging signals and explore their mechanisms.

## 1 Introduction

Avacopan is an orally administered, first‐in‐class small molecule that selectively inhibits the complement 5a receptor (C5aR1) [1]. It is approved for the treatment of severe active antineutrophil cytoplasmic antibody (ANCA)–associated vasculitis, specifically granulomatosis with polyangiitis (GPA) and microscopic polyangiitis (MPA). These rare, potentially life‐threatening autoimmune vasculitides are characterized by necrotizing inflammation of small‐ to medium‐sized blood vessels and typically involve the kidneys, lungs, and other critical organ systems [2]. The pathogenesis involves aberrant neutrophil activation and dysregulated complement signaling via the C5a–C5aR1 axis, leading to chemotaxis, oxidative burst, and endothelial injury. By antagonizing C5aR1, Avacopan interrupts this inflammatory cascade, enabling a targeted therapy that reduces reliance on systemic corticosteroids and their associated toxicity [3].

The pivotal phase III ADVOCATE trial (NCT02994927) provided the primary clinical evidence supporting Avacopan's efficacy. In this multinational, double‐blind, randomized controlled trial involving 331 patients with newly diagnosed or relapsing ANCA‐associated vasculitis, Avacopan was evaluated in combination with standard‐of‐care immunosuppressive therapy, compared with a control group receiving a prednisone taper regimen. At week 26, remission rates with Avacopan were non‐inferior to prednisone (72.3% vs. 70.1%; one‐sided *p* < 0.001), and by week 52, Avacopan demonstrated superiority in achieving sustained remission (65.7% vs. 54.9%; *p* = 0.007). Additionally, Avacopan resulted in greater improvement in renal function, with mean eGFR increases of 7.3 mL/min/1.73 m^2^ compared to 4.1 mL/min/1.73 m^2^ in the prednisone arm at 52 weeks, consistent with post hoc subgroup analyses demonstrating enhanced renal recovery in patients with baseline eGFR ≤ 20 mL/min/1.73 m^2^. Serious adverse events were slightly lower in the Avacopan arm than in controls, supporting its corticosteroid‐sparing safety profile [3].

Upon these findings, the U.S. Food and Drug Administration approved Avacopan in 2021 as an adjunctive treatment for adult patients with severe active ANCA‐associated vasculitis (GPA or MPA), to be used in combination with standard therapy including glucocorticoids, without replacing steroid therapy entirely. Japan approved Avacopan in September 2021 through the Ministry of Health, Labour and Welfare, and the European Commission granted a marketing authorization in January 2022 following a positive CHMP opinion issued in November 2021 [4]. The FDA also granted Avacopan Priority Review and Orphan Drug designations, reflecting its potential to address a significant unmet medical need.

These regulatory milestones positioned Avacopan as a clinically meaningful steroid‐sparing alternative. Its oral formulation, mechanism‐based targeting of C5aR1, and demonstrated tolerability represent substantial advancements in the treatment paradigm of ANCA‐associated vasculitis.

While efficacy and short‐term safety were established in controlled clinical settings, the limited sample size and strict eligibility criteria of pivotal trials inherently restrict generalizability. As a result, rare, delayed, or population‐specific adverse drug reactions (ADRs) may go undetected prior to market authorization. This highlights the critical importance of post‐marketing pharmacovigilance in characterizing Avacopan's safety profile across real‐world populations, especially when administered chronically to immunocompromised patients alongside other immunosuppressives.

The FDA Adverse Event Reporting System (FAERS) serves as a cornerstone of post‐approval safety surveillance in the United States. It collects voluntary reports from healthcare professionals, manufacturers, and consumers, enabling disproportionality analyses to detect whether certain adverse events are reported more frequently than expected. Despite limitations, such as underreporting, variable report quality, and lack of exposure denominators, FAERS remains a valuable tool for early detection of previously unrecognized or emerging safety concerns.

To date, no published pharmacovigilance study has yet systematically assessed Avacopan using spontaneous reporting data sources, representing a significant gap in real‐world evidence since the drug's FDA approval. Recognizing this, our study examines data from Q1 2022 through Q1 2025, deliberately starting after the initial full year post‐approval to ensure stability in reporting patterns following product launch. Using multiple signal detection metrics, we aim to identify statistically significant ADR signals, distinguish between labeled and off‐label events, and provide real‐world evidence to guide clinicians and regulators.

## 2 Materials and Methods

### 2.1 Data Source and Selection Criteria

This study analyzed individual case safety reports (ICSRs) submitted to the United States Food and Drug Administration (FDA) Adverse Event Reporting System (FAERS) covering the period from the first quarter of 2022 (2022Q1) to the first quarter of 2025 (2025Q1). Although Avacopan was approved in the United States in late 2021, the FAERS dataset for 2021 contained only a single report, in which most demographic and outcome information was missing. Therefore, to ensure data integrity and avoid instability associated with very early post‐marketing submissions, the analytical window was restricted to reports from 2022Q1 onward. Data files for each quarter, including DEMO (demographics), DRUG (drug information), REAC (adverse reactions), OUTC (outcomes), RPSR (report source), and THER (therapy timing), were obtained from the FDA's official open‐access platform [5].

To ensure comprehensive capture of relevant cases, a two‐step identification strategy was applied to extract FAERS entries associated with Avacopan. First, both the proprietary name and the generic name were included in a case‐insensitive search of the DRUG file. Second, entries were retained only if Avacopan was coded as the primary suspect (PS) drug, thereby excluding reports in which it was recorded solely as a concomitant or interacting agent. To address potential inconsistencies in drug nomenclature, brand‐to‐generic mapping was cross‐referenced with Structured Product Labeling (SPL) entries in DailyMed to validate naming accuracy.

Reports were excluded if they contained missing or ambiguous drug names, lacked reaction terms, or represented duplicate case identifiers (CASEIDs). Only records submitted after Avacopan's formal market entry and within the specified analytical window were eligible for downstream processing and signal detection.

### 2.2 Data Cleaning and Deduplication

Following data extraction, a standardized preprocessing workflow was applied to enhance data quality and analytical rigor. Duplicate reports were identified using the unique case identifier (CASEID) and the FDA receipt date (FDA_DT), and only the most recent entry for each case was retained in accordance with FDA best practices. If multiple records shared the same CASEID, the entry with the latest FDA_DT was preserved, and earlier submissions were excluded to prevent duplication bias.

To increase the specificity of drug‐event association, the dataset was restricted to cases where Avacopan was designated as the primary suspect drug. Records identifying it as a secondary suspect, concomitant, or interacting agent were omitted, reducing potential confounding from polypharmacy. This step ensured that the detected signals could be more confidently attributed to Avacopan exposure.

Further quality control involved screening for missing or invalid values in key analytical fields. Reports lacking standardized adverse event terms in the REAC file or those with undefined drug names in the DRUG table were excluded. Demographic variables such as age and sex were not required for inclusion in the primary dataset, but were incorporated into subgroup analyses only when explicitly reported.

**FIGURE 1:**
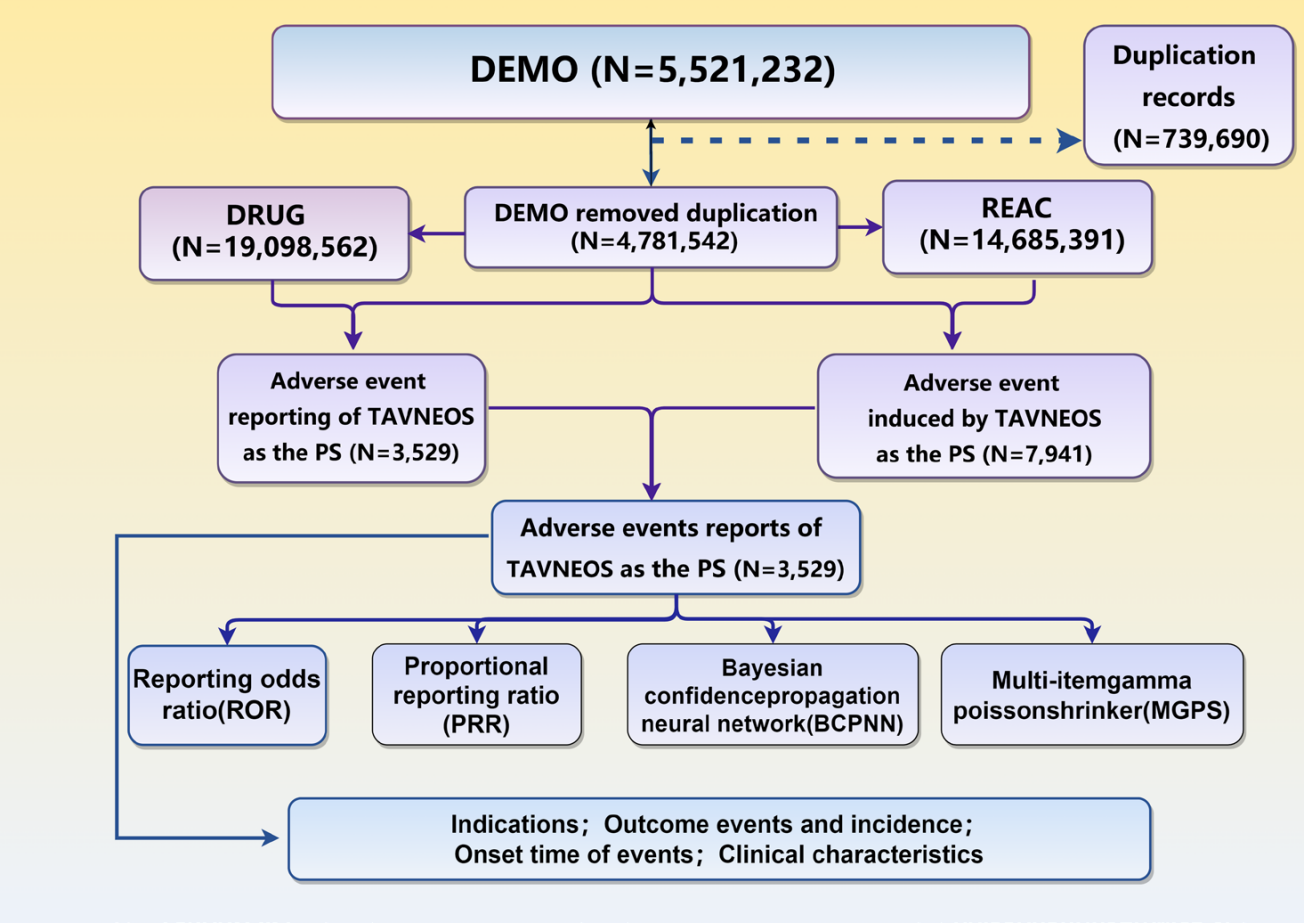
The flowchart illustrating the selection process of our study.

To enable systematic signal aggregation, all Preferred Terms (PTs) were mapped to their corresponding System Organ Classes (SOCs) using the most recent version of the Medical Dictionary for Regulatory Activities (MedDRA) available at the time of analysis. Random spot checks were performed to confirm the accuracy of drug name harmonization and adverse event coding (Figure 1).

### 2.3 Adverse Event Coding and Grouping

Adverse events reported in the FAERS database were classified using the Medical Dictionary for Regulatory Activities (MedDRA), a hierarchical standardized terminology widely adopted for pharmacovigilance analyses. To align with prior preprocessing steps, each reported event was mapped to its corresponding PT, which represents the most granular clinical level in MedDRA. Each PT was then systematically assigned to its associated SOC, the highest MedDRA level grouping related adverse events by organ system or etiology. This two‐tier classification enabled detailed signal detection at the PT level and higher‐level aggregation by SOC to reveal patterns across clinical domains. The MedDRA version used in this study corresponded to the most recent public release available at the time of data extraction, ensuring compliance with prevailing regulatory standards. Adverse event terms were extracted directly from the REAC file using verbatim entries and cross‐checked against the PT dictionary. When multiple PTs were reported in a single case, all entries were retained as separate analytical units to preserve the complexity of each report rather than collapsing them into a single event.

### 2.4 Disproportionality Analysis Methods

To identify safety signals associated with Avacopan in the FAERS database, disproportionality analyses were performed using four established quantitative metrics: Reporting Odds Ratio (ROR), Proportional Reporting Ratio (PRR), Information Component (IC), and Empirical Bayes Geometric Mean (EBGM). Among these, ROR served as the principal method for signal determination due to its interpretability and widespread adoption in pharmacovigilance studies. These methods evaluate whether the observed frequency of a specific drug–event combination exceeds the expected frequency derived from the entire FAERS database, enabling the detection of statistically disproportionate associations [6, 7, 8].

ROR is a frequentist statistic that compares the odds of reporting a particular adverse event with Avacopan to the odds of that event with all other drugs. An association was defined as a positive signal when the lower bound of the 95% confidence interval exceeded 1 and the number of reports (*N*) was at least 3 [9]. PRR examines whether the proportion of a specific adverse event among all events linked to Avacopan is higher than the corresponding proportion for all other drugs [10]. A signal was recognized when PRR ≥ 2, chi‐square ≥ 4, and *N* ≥ 3. IC is a Bayesian metric developed by the Uppsala Monitoring Centre, and a positive signal was defined when the lower 95% credibility limit exceeded 0. EBGM was calculated using the FDA's Multi‐Item Gamma Poisson Shrinker algorithm. A signal was flagged when the lower 90% credibility bound (EB05) exceeded 2 [11, 12].

All computations were conducted in R (v4.3.2) using the *PhViD* and *openEBGM* packages based on two‐by‐two contingency tables derived from co‐occurrence frequencies. Only drug–event pairs with *N* ≥ 3 were retained for analysis. Detailed computational formulas, signal criteria, and parameter specifications are provided in Table S1.

### 2.5 Stratified and Temporal Analyses

To explore demographic and temporal patterns in adverse event reporting, supplementary subgroup analyses were conducted. Avacopan‐related reports were stratified by sex, age and reporter type, using the DEMO and RPSR tables. For each subgroup, ROR values were recalculated independently to assess whether specific signals were disproportionately concentrated in certain populations. Special attention was given to pediatric cases due to limited clinical trial exposure in this group.

Temporal trends were assessed by aggregating the number of reports per calendar quarter from 2022Q1 to 2025Q1. Time series plots were generated to visualize potential changes in reporting frequency over time. These trends were examined descriptively and were not subjected to formal time‐series modeling. All stratified and temporal analyses were exploratory and intended to support contextual interpretation of signal robustness rather than serve as primary evidence of risk differentials.

## 3 Results

### 3.1 Descriptive Characteristics of Reports

Between early 2022 and the first quarter of 2025, a total of 3529 individual case safety reports (ICSRs) involving Avacopan were retrieved from the FAERS database. Demographic completeness varied, with sex reported in 67.2% and age available in 60.4% of all cases.

Among records with specified sex, females accounted for 38.9% (*n* = 1372) and males for 28.3% (*n* = 998), while gender information was missing in 32.8% (*n* = 1159). Regarding age distribution, patients aged 18 to under 65 years represented 28.5% (*n* = 1006) and those 65 years or older comprised 30.2% (*n* = 1066). Reports involving individuals under 18 years of age were infrequent (*n* = 59, 1.7%), and age data were missing in 39.6% (*n* = 1398). Patient weight was rarely captured, being recorded in only 10.7% of reports.

In terms of geographic origin, most ICSRs were submitted from the United States (*n* = 2606, 73.8%), followed by Japan (*n* = 495, 14.0%), while Europe and other regions contributed fewer reports. Regarding case classification, 58.5% of ICSRs were marked as serious (*n* = 2063) based on reporter‐indicated seriousness criteria, such as hospitalization, death, or life‐threatening condition. However, many of these cases lacked complete outcome documentation, making it difficult to ascertain the actual clinical course. Among those with outcome details, death was reported in 9.0% (*n* = 319), hospitalization in 28.1% (*n* = 991), and life‐threatening events in 1.1% (*n* = 40). Non‐serious cases comprised 41.5% (*n* = 1466), although outcome data were frequently missing or incomplete.

Reporter classification indicated that consumers submitted 47.8% of reports, followed by physicians (22.8%), other healthcare professionals (13.9%), and pharmacists (5.7%). Reporter identity was unclassified in 9.8% of ICSRs. Report volume varied by year, peaking in 2023 (*n* = 1545, 43.8%), followed by 2024 (32.6%), 2025Q1 (13.4%), and 2022 (10.2%).

The most commonly reported therapeutic indications were Granulomatosis with Polyangiitis (23.9%), ANCA‐associated vasculitis (18.5%), and Microscopic Polyangiitis (15.2%), aligning with the approved uses of Avacopan. Other reported indications included antifungal prophylaxis (3.8%), non‐specified vasculitis (3.0%), off‐label use (2.4%), and renal or cardiovascular comorbidities.

These baseline characteristics provide critical context for the subsequent signal detection analysis and support subgroup interpretation across patient demographics and clinical scenarios (Table 1).

**TABLE 1:** Clinical characteristics of reports with Avacopanfrom the FAERS database (January 2022 to March 2025).

Characteristics	Avacopan induced AE reports (*n* = 3529)		Case proportion, %
Number of events	Proportion of available data	Case number, *n*	
Gender	67.16%		100.00%
F		1372	38.90%
M		998	28.30%
Missing		1159	32.80%
Age (years)	60.39%		25.60%
< 18		59	1.70%
18 ≤ and < 65		1006	28.50%
≥ 65		1066	30.20%
Missing		1398	39.60%
Weight (Kg)	10.68%		23.50%
< 50 kg		74	2.10%
> 100 kg		26	0.70%
50 ~ 100 kg		237	6.70%
Missing		3192	90.50%
Reported countries	99.09%		100.00%
Australia		16	0.50%
Canada		131	3.70%
Switzerland		22	0.60%
Germany		35	1.00%
Spain		12	0.30%
France		57	1.60%
United Kingdom		113	3.20%
Japan		495	14.00%
Netherlands		10	0.30%
United States		2606	73.80%
Other/missing		32	0.91%
Outcomes			100.00%
Nonserious		1466	41.50%
Serious	^a^	2063	58.50%
Death		319	9.00%
Disability		6	0.20%
Hospitalization		991	28.10%
Life‐threatening		40	1.10%
Other/missing		2173	61.60%
Reporters	90.22%		100.00%
Consumer		1688	47.80%
Health Professional		491	13.90%
Pharmacist		201	5.70%
Physician		804	22.80%
Missing		345	9.80%
Report data year			100.00%
2022		360	10.20%
2023		1545	43.80%
2024		1150	32.60%
2025		474	13.40%
Indications			100.00%
Granulomatosis with polyangiitis		844	23.92%
Anti‐neutrophil cytoplasmic antibody positive vasculitis		653	18.50%
Microscopic polyangiitis		538	15.25%
Antifungal prophylaxis		134	3.80%
Vasculitis		105	2.98%
Off label use		85	2.41%
Arteritis		78	2.21%
Renal Disorder		76	2.15%
Prophylaxis		63	1.79%
Hypertension		50	1.42%
Antineutrophil cytoplasmic antibody		37	1.05%
Antineutrophil cytoplasmic antibody positive		25	0.71%
Prophylaxis against gastrointestinal ulcer		23	0.65%
Constipation		21	0.60%
Dyslipidaemia		19	0.54%
Gastrointestinal disorder prophylaxis		18	0.51%
Osteoporosis		17	0.48%
Insomnia		15	0.43%
Anemia		14	0.40%
Chronic kidney disease		14	0.40%
Other		700	19.84%

^a^ Serious reports are those describing death, life‐threatening illness, hospitalization or prolongation of existing hospitalization, or permanent disability.

### 3.2 Overall Signal Detection and Characterization of On‐Label and Exploratory Events

**FIGURE 2:**
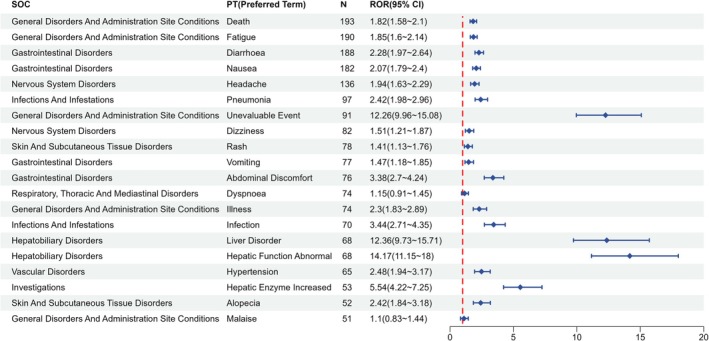
Forest plot of top reported preferred terms (PTs) associated with Avacopan in the FAERS database from January 2022 to March 2025, ranked by report count.

Based on disproportionality analysis of FAERS data from the first quarter of 2022 to the first quarter of 2025, multiple statistically significant adverse event signals associated with Avacopan were identified at the Preferred Term (PT) level. Signal detection was conducted using four established methods—reporting odds ratio (ROR), proportional reporting ratio (PRR), information component (IC), and empirical Bayes geometric mean (EBGM)—with significance defined by conventional thresholds, and the ROR serving as the primary indicator for signal determination. The results are summarized in Table 2, and the strength of each signal is visualized in Figure 2, where PTs are ranked by descending a‐values.

**TABLE 2:** Disproportionality results of adverse events associated with Avacopan in the FAERS database, stratified by system organ class and preferred terms. Adverse events were evaluated using four signal detection methods (ROR, PRR, IC, EBGM). Events marked with a star (★) represent non‐label‐listed adverse events, those not included in the current prescribing information for Avacopan.

SOC	PT (preferred term)	*N*	ROR (95% Cl)	IC (IC025)	PRR (*χ* ^2^)	EBGM (EBGM05)
Blood and lymphatic system disorders	Neutropenia	45	1.99 (1.49–2.67)	0.99 (0.53)	1.99 (22.11)	1.99 (1.48)
	Leukopenia	17	2.96 (1.84–4.77)	1.56 (0.73)	2.96 (22.02)	2.96 (1.84)
	Lymphopenia	6	3.04 (1.36–6.77)	1.6 (0.14)	3.03 (8.18)	3.03 (1.36)
Ear and labyrinth disorders	Deafness	10	3.12 (1.68–5.81)	1.64 (0.51)	3.12 (14.38)	3.12 (1.67)
Gastrointestinal disorders	Diarrhea	188	2.28 (1.97–2.64)	1.17 (0.95)	2.25 (132.09)	2.25 (1.95)
	Nausea	182	2.07 (1.79–2.4)	1.03 (0.81)	2.05 (98.37)	2.05 (1.77)
	Vomiting	77	1.47 (1.18–1.85)	0.56 (0.22)	1.47 (11.62)	1.47 (1.17)
	Abdominal Discomfort	76	3.38 (2.7–4.24)	1.75 (1.37)	3.36 (125.92)	3.35 (2.67)
	Abdominal Pain Upper	49	2.04 (1.54–2.71)	1.03 (0.59)	2.04 (25.93)	2.04 (1.54)
General disorders and administration site conditions	Fatigue	190	1.85 (1.6–2.14)	0.87 (0.65)	1.83 (72.36)	1.83 (1.58)
	Unevaluable Event	91	12.26 (9.96–15.08)	3.59 (3.12)	12.13 (923.93)	12.05 (9.8)
	Illness	74	2.3 (1.83–2.89)	1.19 (0.83)	2.28 (53.56)	2.28 (1.82)
	No Adverse Event	51	1.48 (1.12–1.95)	0.56 (0.15)	1.48 (7.86)	1.48 (1.12)
	Liver Disorder	68	12.36 (9.73–15.71)	3.61 (3.04)	12.26 (699.38)	12.19 (9.59)
Hepatobiliary disorders	Hepatic Function Abnormal	68	14.17 (11.15–18)	3.8 (3.2)	14.06 (818.9)	13.96 (10.98)
	Drug‐Induced Liver Injury	45	8.6 (6.41–11.53)	3.09 (2.44)	8.55 (299.02)	8.52 (6.35)
	Jaundice★	35	18.09 (12.96–25.25)	4.16 (3.12)	18.01 (557.17)	17.85 (12.79)
	Cholestasis★	18	8.19 (5.15–13.02)	3.03 (1.9)	8.18 (112.91)	8.14 (5.12)
	Anti‐Neutrophil Cytoplasmic Antibody Positive Vasculitis	22	46.81 (30.64–71.51)	5.51 (3.34)	46.69 (959.37)	45.56 (29.82)
Immune system disorders	Immunosuppression	6	4.52 (2.03–10.07)	2.17 (0.49)	4.52 (16.38)	4.51 (2.02)
	Pneumonia	97	2.42 (1.98–2.96)	1.27 (0.95)	2.41 (80.1)	2.41 (1.97)
Infections and infestations	Infection	70	3.44 (2.71–4.35)	1.77 (1.38)	3.41 (119.6)	3.41 (2.69)
	Urinary Tract Infection	42	1.86 (1.38–2.52)	0.89 (0.42)	1.86 (16.69)	1.86 (1.37)
	Sepsis	30	2.46 (1.72–3.52)	1.29 (0.71)	2.45 (25.86)	2.45 (1.71)
	Influenza	26	1.55 (1.06–2.28)	0.63 (0.05)	1.55 (5.09)	1.55 (1.05)
	Hepatic Enzyme Increased	53	5.54 (4.22–7.25)	2.46 (1.95)	5.5 (195.05)	5.49 (4.19)
Investigations	Blood Pressure Increased	36	1.79 (1.29–2.48)	0.83 (0.33)	1.78 (12.41)	1.78 (1.28)
	Blood Creatinine Increased	28	3.99 (2.75–5.78)	1.99 (1.31)	3.98 (62.37)	3.97 (2.74)
	Liver Function Test Increased	28	7.84 (5.41–11.38)	2.96 (2.12)	7.82 (165.88)	7.79 (5.37)
	Alanine Aminotransferase Increased	25	4.34 (2.93–6.43)	2.11 (1.37)	4.33 (63.93)	4.32 (2.92)
	Decreased Appetite	47	1.54 (1.16–2.06)	0.62 (0.19)	1.54 (8.93)	1.54 (1.16)
Metabolism and nutrition disorders	Fluid Retention	13	2.45 (1.42–4.21)	1.29 (0.37)	2.44 (11.07)	2.44 (1.42)
	Increased Appetite	12	5.27 (2.99–9.29)	2.39 (1.18)	5.26 (41.31)	5.25 (2.98)
	Steroid Diabetes	9	32.91 (17.02–63.64)	5.01 (2.05)	32.88 (273.3)	32.32 (16.71)
	Hypervolaemia	6	4 (1.79–8.91)	2 (0.39)	4 (13.46)	3.99 (1.79)
	Headache	136	1.94 (1.63–2.29)	0.94 (0.68)	1.92 (60.45)	1.92 (1.62)
Nervous system disorders	Dizziness	82	1.51 (1.21–1.87)	0.59 (0.26)	1.5 (13.88)	1.5 (1.21)
	Paraesthesia	28	1.6 (1.1–2.32)	0.67 (0.11)	1.6 (6.23)	1.59 (1.1)
	Cerebral Infarction	6	2.67 (1.2–5.95)	1.42 (0.01)	2.67 (6.25)	2.67 (1.2)
	Insomnia	48	1.76 (1.32–2.33)	0.81 (0.37)	1.75 (15.54)	1.75 (1.32)
Psychiatric disorders	Renal Impairment	43	3.85 (2.85–5.19)	1.94 (1.41)	3.83 (89.96)	3.83 (2.83)
Renal and urinary disorders	Renal Disorder	28	5.12 (3.53–7.43)	2.35 (1.62)	5.11 (92.31)	5.1 (3.51)
	Renal Failure	28	2.33 (1.61–3.37)	1.21 (0.61)	2.32 (21.09)	2.32 (1.6)
	End Stage Renal Disease	11	6.35 (3.51–11.48)	2.66 (1.3)	6.34 (49.36)	6.33 (3.5)
	Proteinuria	9	3.4 (1.77–6.54)	1.76 (0.54)	3.4 (15.21)	3.39 (1.76)
	Epistaxis	20	2.63 (1.7–4.08)	1.39 (0.65)	2.63 (20.16)	2.63 (1.69)
Respiratory, thoracic and mediastinal disorders	Lung Disorder	19	3 (1.91–4.71)	1.58 (0.8)	3 (25.25)	2.99 (1.91)
	Interstitial Lung Disease	19	3.09 (1.97–4.85)	1.62 (0.83)	3.08 (26.72)	3.08 (1.96)
	Pulmonary Embolism	14	1.84 (1.09–3.11)	0.88 (0.05)	1.84 (5.34)	1.84 (1.09)
	Haemoptysis	10	3.72 (2–6.92)	1.89 (0.7)	3.72 (19.84)	3.71 (2)
	Rash	78	1.41 (1.13–1.76)	0.49 (0.15)	1.4 (9.09)	1.4 (1.12)
Skin and subcutaneous tissue disorders	Alopecia★	52	2.42 (1.84–3.18)	1.27 (0.83)	2.41 (43.05)	2.41 (1.83)
	Yellow Skin	3	7.28 (2.34–22.63)	2.86 (0.05)	7.28 (16.18)	7.25 (2.33)
	Hypertension	65	2.48 (1.94–3.17)	1.3 (0.91)	2.47 (56.86)	2.47 (1.93)
Vascular disorders	Vasculitis	27	17.37 (11.88–25.38)	4.1 (2.89)	17.31 (411.16)	17.16 (11.74)
	Thrombosis★	20	2.32 (1.49–3.59)	1.21 (0.49)	2.31 (14.89)	2.31 (1.49)
	Granulomatosis With Polyangiitis	18	80.93 (50.46–129.81)	6.27 (3.27)	80.75 (1358.44)	77.41 (48.26)
	Deep Vein Thrombosis★	14	3.3 (1.95–5.58)	1.72 (0.77)	3.3 (22.35)	3.29 (1.95)

**FIGURE 3:**
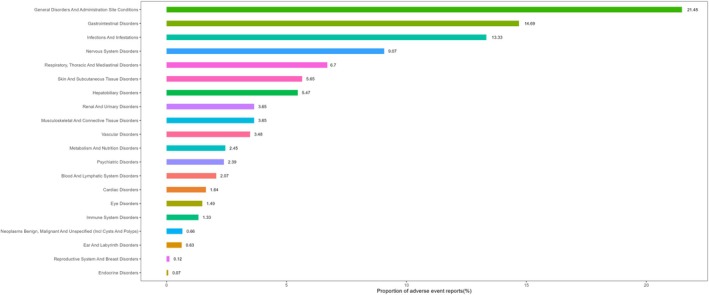
Distribution of Avacopan‐related adverse event reports by System Organ Class (SOC).

The corresponding System Organ Class (SOC) distribution, displayed in Figure 3, demonstrates that most high‐signal events were clustered in hepatobiliary disorders, general disorders and administration site conditions, infections, gastrointestinal disorders, and nervous system disorders. These SOCs are aligned with previously described target organ systems for complement modulation and immunosuppressive therapy.

Comparison with the most recent FDA‐approved prescribing information revealed that a substantial proportion of detected PTs corresponded with labeled adverse events. Specifically, signal‐positive PTs such as hepatocellular injury, elevated transaminases, hypertension, nausea, vomiting, diarrhea, fatigue, headache, and infectious events including pneumonia and urinary tract infections were all listed within the labeling under ‘Adverse Reactions’ or ‘Warnings and Precautions’, reaffirming the external consistency of post‐marketing observations with pivotal trial data.

**FIGURE 4:**
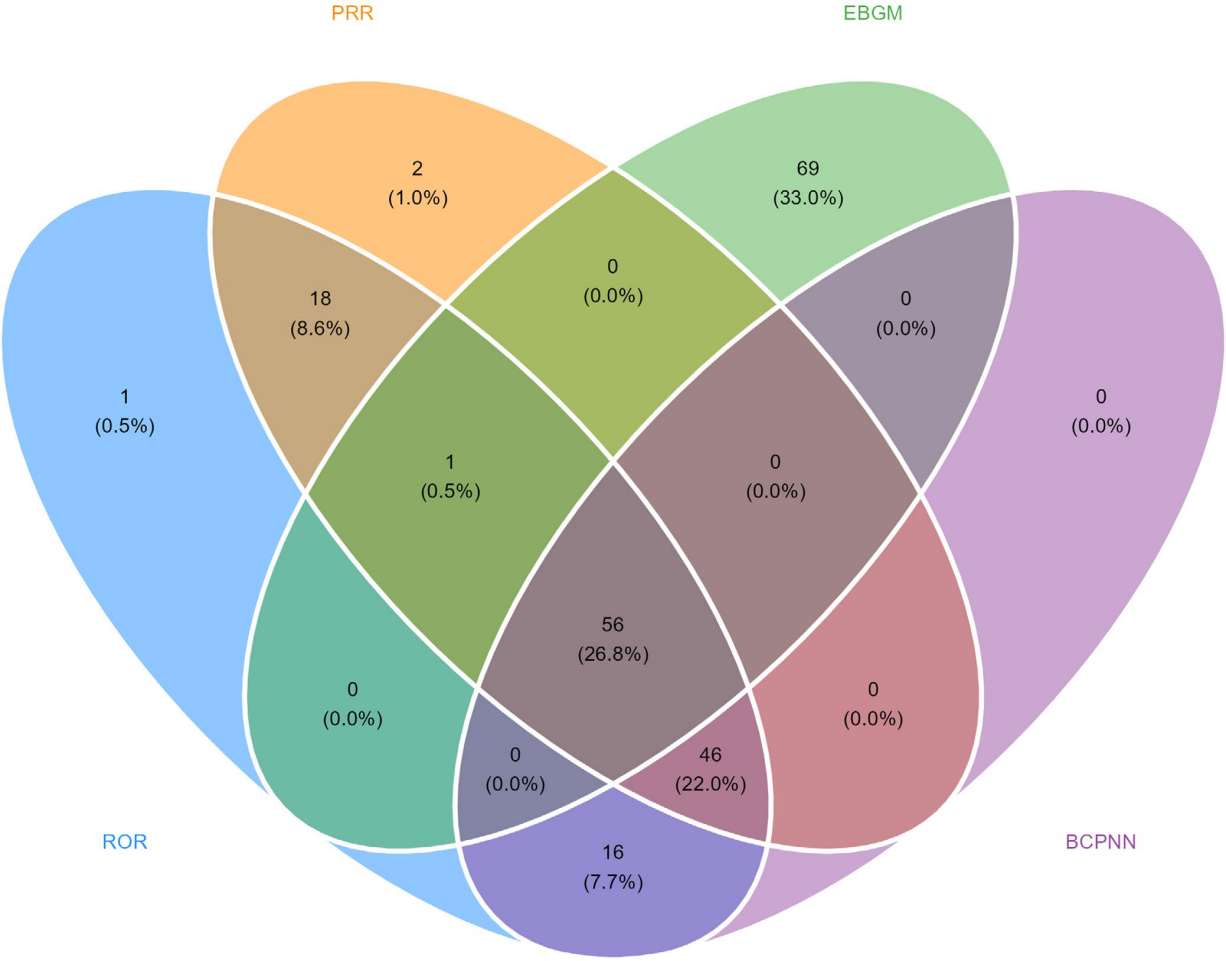
Venn diagram illustrating the overlap of positive adverse event signals detected by four disproportionality methods: ROR, PRR, EBGM, and BCPNN.

In contrast, several disproportionate signals were identified for PTs not documented in any section of the Avacopan label. These included alopecia, venous thromboembolic events (e.g., deep vein thrombosis), cholestatic hepatic reactions (e.g., jaundice, cholestasis), and electrolyte abnormalities, none of which were mentioned in the boxed warning, clinical trial adverse reaction tables, or post marketing experience sections. These potentially novel signals are illustrated in Figure 4, which highlights their separation from labeled events.

Notably, many of these exploratory PTs were associated with serious outcomes in the FAERS reports, particularly those involving thromboembolic and hepatobiliary events. Although causality cannot be inferred from spontaneous reporting alone, the emergence of statistically elevated signals for unlabeled events, particularly when associated with severity, warrants closer regulatory and clinical attention.

Overall, the findings confirm the presence of known adverse reactions in real‐world settings and simultaneously highlight several unlisted but potentially meaningful safety signals, suggesting areas for enhanced surveillance, regulatory scrutiny, or future label refinement. The integration of quantitative disproportionality metrics and outcome seriousness provides a structured foundation for ongoing pharmacovigilance beyond the scope of clinical trials.

### 3.3 Subgroup and Temporal Distribution Analysis

**FIGURE 5:**
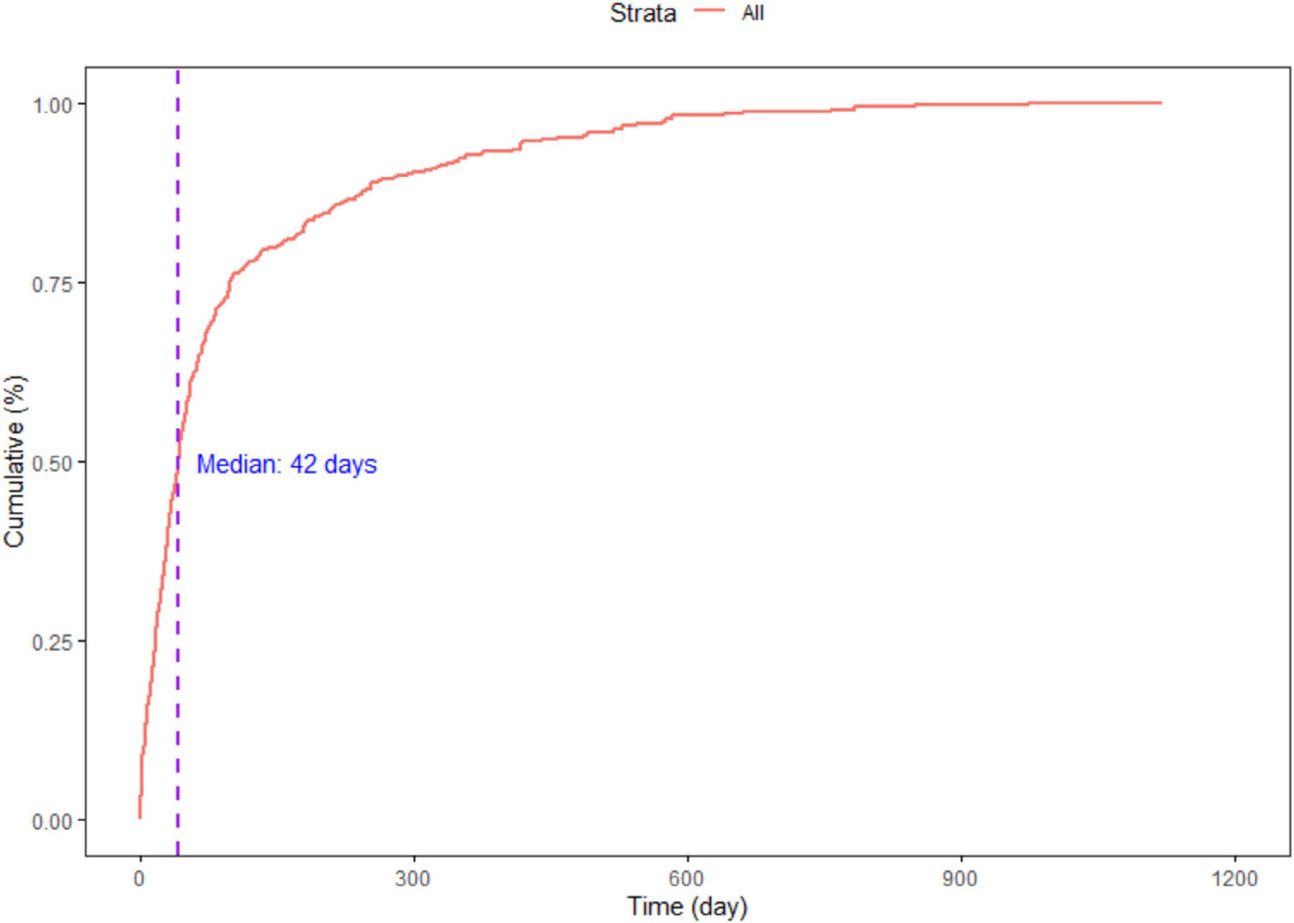
Cumulative incidence curve showing the overall time‐to‐onset distribution of Avacopan‐associated adverse events, with a median onset time of 42 days.

The cumulative onset patterns of adverse events (AEs) following Avacopan exposure exhibited notable heterogeneity across demographic subgroups and reporter types. As visualized in Figure 5, AE reports demonstrated a steep initial rise, with the majority of events clustering within the early phase of treatment. The estimated median time to onset was 42 days, indicating that a substantial proportion of AEs occurred within the first several weeks after treatment initiation.

**FIGURE 6:**
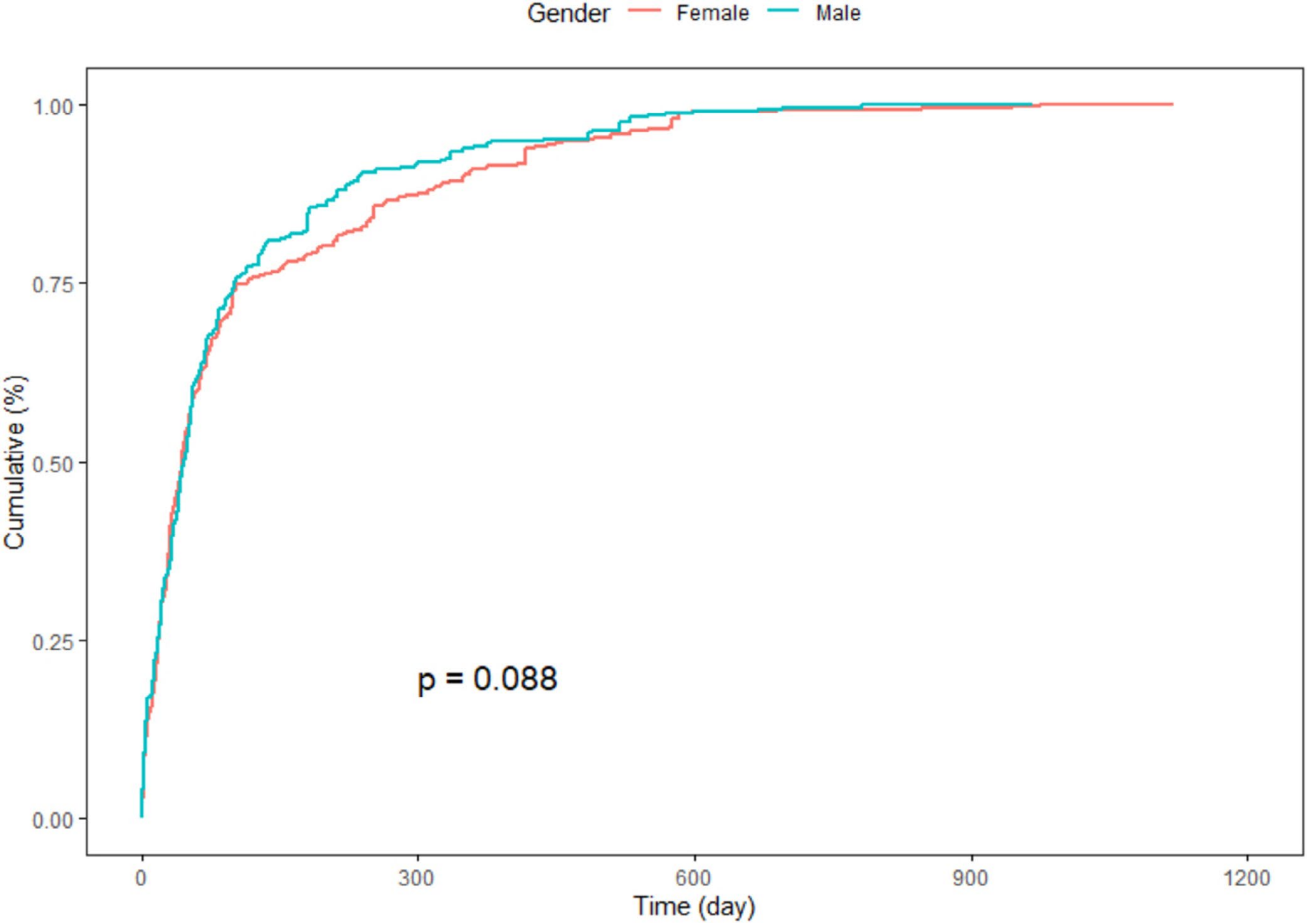
Time‐to‐onset profiles between male and female patients show no statistically significant variation, although males exhibit a slightly faster cumulative reporting pattern.

**FIGURE 7:**
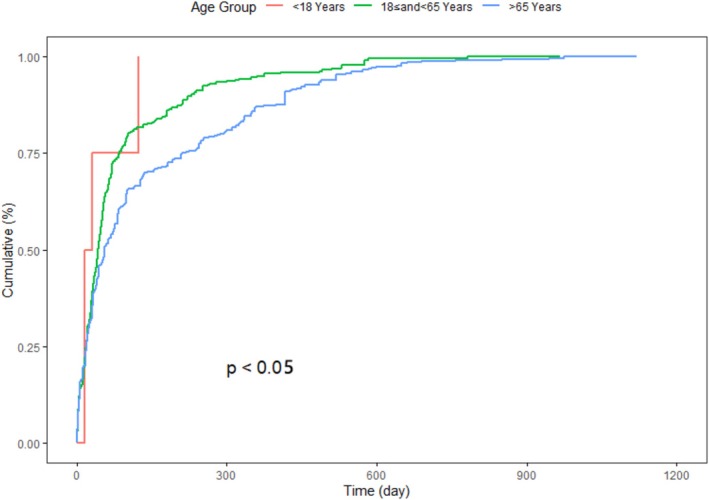
Stratified Kaplan–Meier curves illustrate significant differences in time‐to‐onset of adverse events across age groups.

Sex‐based stratification showed a mildly slower accumulation of AEs in female patients compared to males, although the difference was not statistically significant (*p* = 0.088), as shown in Figure 6. In contrast, age‐stratified Kaplan–Meier analysis revealed more distinct temporal patterns. AE onset in individuals under 18 years of age was tightly clustered within the initial treatment period, while patients aged 65 years and older exhibited a more gradual cumulative increase, as seen in Figure 7.

Nevertheless, interpretation of the pediatric curve warrants caution due to the limited number of cases in this subgroup, which may artifactually accentuate the steep and flat pattern of the curve.

**FIGURE 8:**
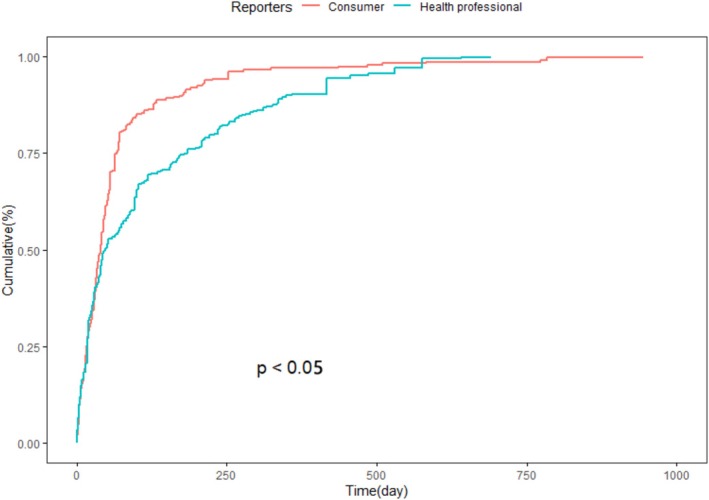
Report source–stratified KM curves reveal earlier reporting of adverse events by consumers compared to healthcare professionals.

Further analysis based on the source of report submissions uncovered a marked difference in the temporal spread of AEs. Reports from consumers were associated with an earlier median onset time and a steeper cumulative curve, whereas those submitted by healthcare professionals displayed a more distributed temporal profile, as illustrated in Figure 8 (*p* < 0.05). This divergence could reflect differing thresholds for AE recognition, attribution, or reporting urgency between lay reporters and medical professionals.

**FIGURE 9:**
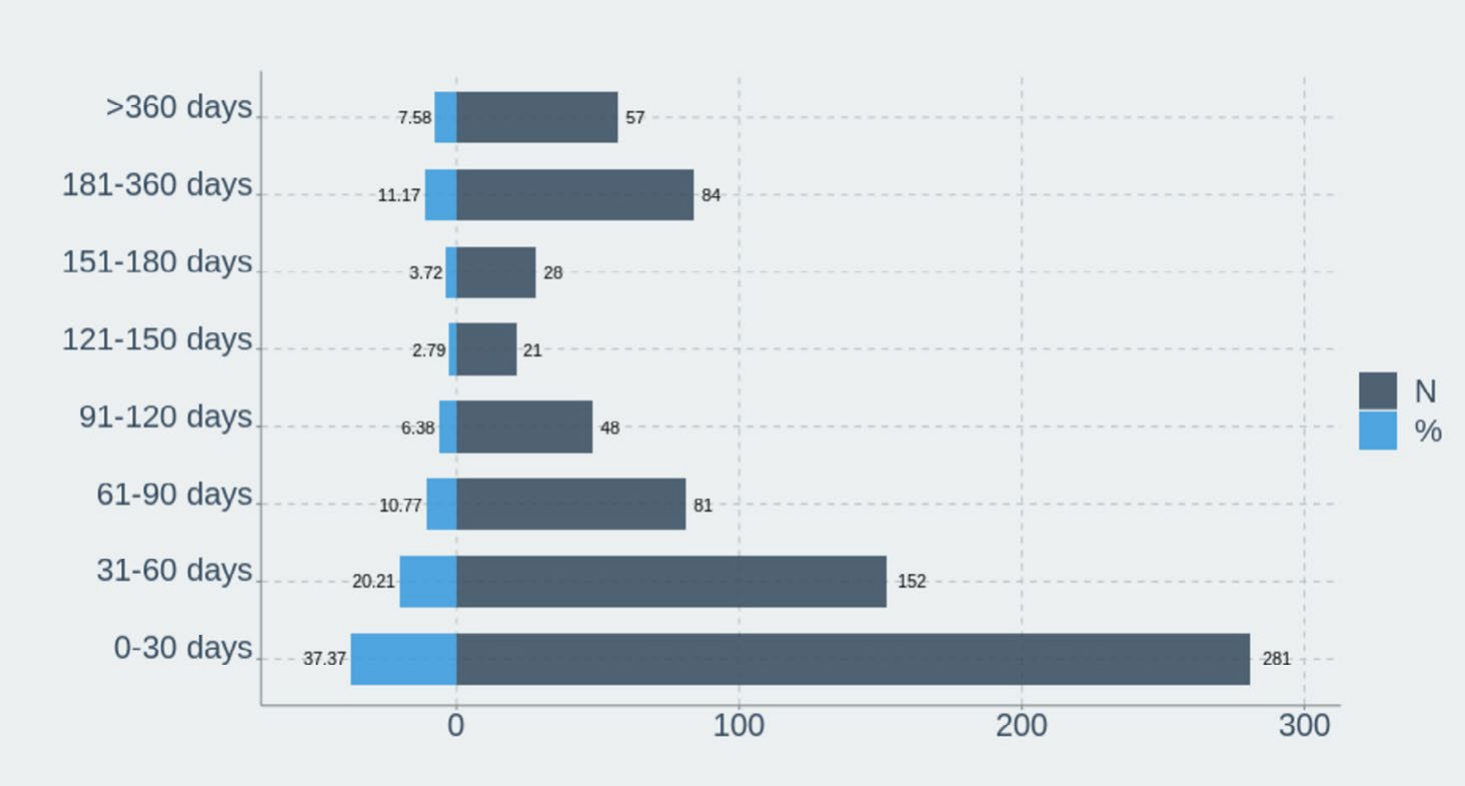
Distribution of adverse event onset times following Avacopan exposure, showing that over half occurred within the first 60 days, with 37.4% arising in the initial month.

To complement the cumulative analysis, AE onset was also examined using a dual‐axis histogram, stratifying frequency by time interval. The Figure 9, demonstrated that over one‐third of AEs were reported within the first 30 days of exposure. Beyond 90 days, AE frequencies declined markedly, suggesting a temporal concentration of risk in the early post‐initiation period.

In aggregate, these findings highlight the need for intensified pharmacovigilance during the early treatment phase, particularly in younger and older populations. Biological susceptibility, variable clinical course recognition, and differences in reporting behavior likely interact to shape the observed temporal landscape. These dynamics should be considered when formulating monitoring protocols or regulatory recommendations.

## 4 Discussion

### 4.1 Overview of Detected Safety Signals

Our analysis identified multiple statistically significant AE signals associated with Avacopan based on disproportionality calculations applied to FAERS data spanning 2022–2025. Although Avacopan received U.S. approval in 2021, only one FAERS report was submitted during that year, and most demographic and clinical information in that report was incomplete. Therefore, data collection was initiated from 2022 to ensure analytical stability and data completeness, minimizing noise from sporadic early post‐marketing submissions. Signal detection was performed using four independent algorithms. The convergence of results across methods enhanced confidence in the associations and reduced methodological bias.

A substantial proportion of signal‐positive preferred terms (PTs) clustered within hepatobiliary, gastrointestinal, vascular, infectious, and nervous system domains, which correspond to the expected effects of C5a receptor blockade on complement‐mediated inflammation. Notably, several highly enriched PTs, including fatigue, nausea, liver enzyme abnormalities, hypertension, pneumonia, and urinary tract infections, are listed in the current Avacopan label, confirming the consistency between clinical trials and post‐marketing reports [13].

In addition to these labeled events, certain PTs demonstrated elevated disproportionality metrics across all detection methods yet remain absent from the official prescribing information. These include alopecia, venous thromboembolism, and cholestatic liver injury such as jaundice and cholestasis. Although infrequent in pre‐approval studies, their recurrent appearance in real‐world data may reflect delayed onset, under detection during trial monitoring, or susceptibility in patients with complex comorbidities [14, 15].

Importantly, these exploratory PTs were disproportionately associated with serious clinical outcomes, including hospitalization and life‐threatening events. While causality cannot be confirmed using FAERS, the signal strength and consistency suggest these may represent underrecognized risks. Additional prospective and mechanistic investigations are needed before formal incorporation into clinical guidelines or regulatory communications.

### 4.2 Concordance With FDA Label and Clinical Trials

The AE spectrum identified in FAERS largely mirrored the safety findings of the ADVOCATE trial and the current U.S. prescribing information. Hepatic laboratory abnormalities, infections such as pneumonia and urinary tract infections, hypertension, gastrointestinal symptoms including nausea, vomiting, and diarrhea, as well as fatigue and headache, all demonstrated statistically elevated signal values, aligning with the most frequently reported treatment‐emergent events in clinical trials. This concordance supports the external generalizability of pre‐approval data to real‐world practice and affirms the current label's precautionary language [16].

However, the magnitude of hepatobiliary signals in FAERS was more prominent than in the ADVOCATE trial, where liver enzyme elevations were generally mild and manageable. In contrast, post‐marketing data included broader hepatobiliary terms such as cholestatic jaundice and liver injury, many involving hospitalization or other serious outcomes. This disparity may reflect a wider clinical spectrum, including patients with baseline hepatic comorbidities or exposure to additional hepatotoxic agents, as well as longer observation periods not captured in controlled trials [4, 13].

Infectious events also remained consistently overrepresented, which is consistent with the immunomodulatory nature of C5a receptor inhibition and the routine use of Avacopan with other immunosuppressants. The continued prominence of pneumonia and urinary tract infections in both trial and post‐marketing contexts highlights the need for vigilant infection monitoring in clinical settings, especially among older or immunocompromised patients.

Several signals observed in our analysis, particularly alopecia, venous thromboembolism, and cholestatic liver injury, are not currently listed in the Avacopan label and were not emphasized in the pivotal trial or its extension. Although spontaneous reports cannot establish causality, the signal strength across multiple algorithms supports further clinical attention. In particular, thrombotic events such as deep vein thrombosis and pulmonary embolism may be influenced by underlying disease activity or concurrent glucocorticoid therapy, but still warrant prospective evaluation [17].

Reassuringly, typical steroid‐associated toxicities, including hyperglycemia and osteoporosis, were not disproportionately represented in FAERS. This observation is consistent with recent open‐label studies suggesting that Avacopan preserves metabolic and endocrine stability over extended use.

In summary, the observed concordance between FAERS findings, trial data, and existing labeling affirms the robustness of the known safety profile, while the emergence of additional exploratory signals underscores the importance of ongoing surveillance and may inform future updates to product labeling.

### 4.3 Potential New Safety Signals and Clinical Plausibility

The FAERS‐based analysis provides additional safety insights that extend current understanding of Avacopan‐associated risks and highlight the need for sustained vigilance in clinical use. The confirmation of labeled events such as hepatotoxicity, infections, and gastrointestinal intolerance underscores the importance of routine monitoring, particularly liver function and infection surveillance, throughout treatment [18]. These precautions are especially critical for patients receiving concomitant immunosuppressive therapies, where cumulative immune suppression may increase susceptibility to hepatic or infectious complications.

The emergence of unexpected signals, including alopecia, thromboembolic events, and cholestatic liver reactions, although requiring cautious interpretation, points to potential off‐label toxicities that may have gone undetected in earlier trials.

Among these, venous thromboembolism (VTE) appears biologically plausible. Patients with ANCA‐associated vasculitis have an intrinsically elevated baseline risk of VTE, and concurrent or recent glucocorticoid exposure further increases thrombotic risk through endothelial activation and altered coagulation balance. Experimental studies have shown that C5a signaling via C5aR1 can promote tissue factor expression, neutrophil extracellular trap formation, and endothelial injury, providing a mechanistic link between complement dysregulation and thrombosis. These observations underscore the need for vigilant monitoring during early treatment and in patients with additional prothrombotic risk factors [19, 20].

Alopecia, although less clinically severe, also presents a biologically coherent signal. Hair‐follicle immune privilege disruption and complement‐mediated inflammation have been implicated in autoimmune hair loss. Cutaneous cells, including keratinocytes and mast cells, express C5aR1, and dysregulated complement activity may contribute to perifollicular inflammation [21, 22]. While direct causal evidence linking Avacopan to alopecia is lacking, dermatologic surveillance may be advisable if progressive hair loss occurs.

Cholestatic liver reactions, though less frequently reported, warrant particular attention because complement components play a recognized role in hepatic immune injury. Experimental data indicate that C5a–C5aR1 signaling in Kupffer cells and cholangiocytes amplifies inflammatory cytokine release and bile acid–induced oxidative stress, which may predispose individuals to cholestatic injury. Given that Avacopan modulates the C5a–C5aR1 axis, a plausible hypothesis is that compensatory complement activation or altered hepatocellular immune signaling could contribute to this pattern [23, 24]. While the limited number of reports precludes definitive interpretation, liver enzyme monitoring remains advisable even in the absence of overt hepatotoxicity symptoms.

These signals should be viewed as hypothesis‐generating findings rather than definitive safety outcomes. Further validation using controlled pharmacoepidemiologic designs and mechanistic investigations focusing on complement–coagulation interactions may help clarify drug‐attributable risks.

In practice, these observations may support more detailed baseline assessments and individualized pharmacovigilance plans. For instance, patients with thrombotic predisposition or those co‐treated with corticosteroids or cytotoxic agents may benefit from targeted risk mitigation strategies before initiating Avacopan [18].

Population‐specific differences further refined the safety profile. Among elderly patients, the delayed accumulation of adverse events suggests the need for prolonged monitoring even in cases of apparent clinical stability. In contrast, the clustering of early‐onset events among pediatric reports, despite their limited number, may indicate heightened pharmacodynamic sensitivity. This observation could support adjustments to dosing strategies or necessitate more frequent safety assessments during the initial treatment phase [3].

Differences in AE reporting timelines between consumers and healthcare professionals also carry implications. Consumer reports tended to capture earlier onset, whereas clinician‐submitted cases were more delayed and potentially more severe. This discrepancy underscores the value of incorporating patient‐reported symptoms into clinical assessments, even when not immediately substantiated by diagnostic evidence. Rather than relying solely on prescriber‐initiated documentation, a more responsive and patient‐engaged pharmacovigilance strategy may be necessary to capture early warning signs and adapt monitoring intensity based on individual susceptibility.

### 4.4 Temporal and Population‐Level Safety Patterns

Our study revealed temporal and demographic heterogeneity in adverse event (AE) patterns associated with Avacopan, providing further granularity to the safety signals identified in aggregate analyses. Over one‐third of AEs occurred during the first 30 days, with the cumulative incidence curve rising sharply during this early exposure window. This clustering highlights the early treatment phase as a critical period for pharmacovigilance, particularly for hepatotoxicity, infections, and gastrointestinal intolerance, which were disproportionately represented.

The timing and frequency of AE onset varied across demographic strata. In patients aged 65 years or older, AE accumulation was slower and more prolonged, indicating that some toxicities may emerge later or require sustained exposure. This underscores the need for extended monitoring in older adults, especially those with impaired hepatic or renal function. Conversely, among pediatric reports, although the number was limited, early clustering of AEs raised concerns about pharmacodynamic sensitivity, warranting further investigation in age‐specific pharmacokinetic studies [25, 26].

Reporting behavior also varied between consumers and healthcare professionals. Consumer‐submitted reports more often reflected AEs in the early weeks of treatment, while clinician‐submitted cases accumulated more gradually. This divergence may reflect differences in symptom awareness, thresholds for concern, or timing of clinical encounters, emphasizing the importance of incorporating both perspectives in post‐marketing surveillance [27].

### 4.5 Study Limitations and Future Directions

While this study offers a detailed characterization of Avacopan‐associated safety signals using FAERS data, several methodological constraints inherent to spontaneous reporting systems merit attention. First, the voluntary nature of FAERS submissions results in substantial underreporting, especially for non‐serious or delayed adverse events [28]. In addition, reporting may be influenced by media attention, regulatory actions, or product label changes, potentially distorting signal patterns.

FAERS also lacks exposure denominators, precluding estimation of absolute incidence rates and limiting cross‐drug or cross‐population comparisons. Elevated report counts may reflect higher drug use rather than true pharmacologic risk. The absence of key clinical details, including comorbidities, laboratory results, or treatment duration, further impedes causal attribution. Incomplete data in core variables such as age or onset time reduce the reliability of subgroup and temporal analyses, as noted previously.

Moreover, disproportionality metrics like ROR and IC capture reporting imbalances rather than causality. These findings should be viewed as hypothesis‐generating and require confirmation in studies with more robust methodology [6, 29].

Future work should validate key signals in other pharmacovigilance systems such as EudraVigilance or JADER to assess geographic consistency. Prospective observational studies embedded in real‐world registries are needed to quantify incidence, identify risk factors, and clarify clinical outcomes, particularly for hepatobiliary and thrombotic events. Mechanistic investigations should also explore how C5aR1 blockade influences immune and vascular responses, especially in age‐ or disease‐vulnerable populations.

Combining spontaneous reporting data with longitudinal and mechanistic evidence will be essential for refining the safety profile of Avacopan and guiding regulatory and clinical decision‐making.

## 5 Conclusion

Our study provides an initial characterization of Avacopan's post‐marketing safety profile, revealing both labeled and potentially unrecognized adverse event signals. While hepatotoxicity and infections remain consistent with prior clinical data, unexpected signals such as thromboembolic and dermatologic events warrant further scrutiny. Temporal clustering of early‐onset reactions and subgroup‐specific differences underscore the importance of targeted monitoring strategies. Although subject to limitations inherent to spontaneous reporting systems, these findings offer valuable real‐world insight and support the need for continued surveillance and confirmatory studies in broader patient populations.

## Author Contributions

**Guojun Liang, Qiong Liu and Xiaofang Liu:** conception and design. **Guojun Liang, Yang Song and Qiong Liu:** collection and assembly of data. **Qiong Liu, Mengge Gao and Bo Yang:** data analysis and interpretation. All authors Manuscript writing. All authors Final approval of manuscript.

## Disclosure

During the preparation of this work the author(s) used DeepL (https://www.deepl.com/en/write) and Youdao (https://fanyi.youdao.com/#/AITranslate) in order to improve readability. After using this tool/service, the author(s) reviewed and edited the content as needed. The author(s) take(s) full responsibility for the content of the publication.

## Ethics Statement

This study involves a secondary analysis of publicly accessible summary statistics and does not necessitate explicit ethical approval.

## Conflicts of Interest

The authors declare no conflicts of interest.

## Supporting information


**Table S1:** Summarizes the computational principles and signal detection criteria for the four disproportionality algorithms (ROR, PRR, IC, and EBGM) applied in this study.

## Data Availability

The data related to this study has been stored in a publicly accessible repository (https://fis.fda.gov/extensions/FPD‐QDE‐FAERS/FPD‐QDE‐FAERS.html). All data produced or examined in this article is available within the published paper and its Supporting Information—S1 files.
