# Supplementary material for: A Real‐World Disproportionality Analysis of Avacopan in Anti‐Neutrophil Cytoplasmic Antibodies Associated Vasculitis: Insights From FDA Adverse Event Reporting System
Source: Pharmacol Res Perspect. 2025 Nov 17;13(6):e70194. doi: 10.1002/prp2.70194 (PMC12620843; doi:10.1002/prp2.70194)
Supplement: Supplementary file 1 — Table S1: Summarizes the computational principles and signal detection criteria for the four disproportionality algorithms (ROR, PRR, IC, and EBGM) applied in this study. [file PRP2-13-e70194-s001.docx]

**Supplementary Tables 1.Two-by-two contingency table for disproportionality analysis.**

| **Item** | **Target adverse events reported** | **Other adverse events reported** | **Total** |
| --- | --- | --- | --- |
| **Target drugs** | a | b | a + b |
| **Other drugs** | c | d | c + d |
| **Total** | a + c | b + d | a + b + c + d |

**The principles of disproportionate measurement and the criteria for signal detection.**

| Method | Calculation formula | ﻿Criteria |
| --- | --- | --- |
| ROR | $ROR=\frac{a / c}{b / d}$ | a ≥ 3  95%CI (lower limit) > 1 |
|  | $SE(lnROR)=\sqrt{\frac{1}{a}+\frac{1}{b}+\frac{1}{c}+\frac{1}{d}}$ |  |
|  | $95\%CI= e^{\ln\left( ROR \right)\pm1.96se}$ |  |
| PRR | $PRR=\frac{a / (a+b)}{c / (c+d)}$ | a ≥ 3  PRR ≥ 2  95%CI (lower limit) > 1 |
|  | $SE(lnPRR)=\sqrt{\frac{1}{a}-\frac{1}{a+b}+\frac{1}{c}-\frac{1}{c+d}}$ |  |
|  | $95\%CI= e^{\ln\left( PRR \right)\pm1.96se}$ |  |
|  | $\chi2 =\frac{{(ad-bc)}^{2}(a+b+c+d)}{( a+b)(a+c)(c+d)(b+d)}$ | a ≥ 3  PRR ≥ 2  $\chi2\geq4$ |
| BCPNN | IC=${log}_{2}\frac{p(x,y)}{p(x)p(y)}={log}_{2}\frac{a(a+b+c+d)}{(a+b)(a+c)}$ | IC025>0 |
|  | E(IC)=${log}_{2}\frac{(a+\gamma11)(a+b+c+d+\alpha)(a+b+c+d+\beta)}{（a+b+c+d+\gamma）(a+b+\alpha1)(a+c+\beta1)}$ |  |
|  | $V\left( IC \right)=\frac{1}{{(ln2)}^{2}}\{\left[ \frac{\left( a+b+c+d \right)-a+\gamma-\gamma11}{\left( a+\gamma11 \right)\left( 1+a+b+c+d+\gamma\right)} \right]+\left[ \frac{\left( a+b+c+d \right)-\left( a+b \right)+\alpha-\alpha1}{\left( a+b+\alpha1 \right)\left( 1+a+b+c+d+\alpha\right)} \right]+\left[ \frac{\left( a+b+c+d \right)-\left( a+c \right)+\beta-\beta1}{\left( a+c+\beta1 \right)\left( 1+a+b+c+d+\beta\right)} \right]\}$ |  |
|  | $\gamma=\gamma11\frac{(a+b+c+d+\alpha)(a+b+c+d+\beta)}{(a+b+\alpha1)(a+c+\beta1)}$ |  |
|  | *IC-2SD=E(IC)-2*$\sqrt{V(IC)}$  $\alpha1=\beta1=1；\alpha=\beta=2；\gamma11=1$ |  |
| EBGM | $EBGM=\frac{a(a+b+c+d)}{\left( a+c \right)(a+b)}$ | EBGM05>2 |
|  | $SE(lnEBGM)=\sqrt{\frac{1}{a}+\frac{1}{b}+\frac{1}{c}+\frac{1}{d}}$ |  |
|  | $95\%CI= e^{\ln\left( EBGM \right)\pm1.96se}$ |  |
